# CCL21-CCR7 signaling promotes microglia/macrophage recruitment and chemotherapy resistance in glioblastoma

**DOI:** 10.1007/s00018-023-04788-7

**Published:** 2023-06-14

**Authors:** Luiz Henrique Geraldo, Celina Garcia, Yunling Xu, Felipe Saceanu Leser, Izabella Grimaldi, Eduardo Sabino de Camargo Magalhães, Joost Dejaegher, Lien Solie, Cláudia Maria Pereira, Ana Helena Correia, Steven De Vleeschouwer, Bertrand Tavitian, Nathalie Henriques Silva Canedo, Thomas Mathivet, Jean-Leon Thomas, Anne Eichmann, Flavia Regina Souza Lima

**Affiliations:** 1 Laboratório de Biologia das Células Gliais, Instituto de Ciências Biomédicas, Universidade Federal do Rio de Janeiro (UFRJ), Rua César Pernetta, 1.766, Cidade Universitária da UFRJ, Rio de Janeiro, RJ 21949-590 Brazil; 2 Université de Paris, PARCC, INSERM, 75015 Paris, France; 3 Laboratory of Experimental Neurosurgery and Neuroanatomy, Department of Neurosciences, KU Leuven, Leuven, Belgium; 4 Department of Neurosurgery, KU Leuven, Leuven, Belgium; 5 Faculdade de Odontologia, Universidade Federal do Rio de Janeiro, Rio de Janeiro, 21949-590 Brazil; 6 Departmento de Patologia, Faculdade de Medicina, Hospital Universitário Clementino Fraga Filho, Universidade Federal do Rio de Janeiro, Rio de Janeiro, Brazil; 7 Université Pierre et Marie Curie Paris 06 UMRS1127, Sorbonne Université, Paris, France; 8 Department of Neurology, Yale University School of Medicine, New Haven, CT 06510-3221 USA; 9 Present Address: Department of Internal Medicine, Cardiovascular Research Center, Yale University School of Medicine, New Haven, CT 06510-3221 USA; 10 Present Address: Department of Cellular and Molecular Physiology, Yale University School of Medicine, New Haven, CT 06510-3221 USA

**Keywords:** Tumor-associated microglia and macrophages, Chemokines, Vascular dysmorphia, Tumor microenvironment, Temozolomide

## Abstract

Glioblastoma (GBM) is the most common and fatal primary tumor of the central nervous system (CNS) and current treatments have limited success. Chemokine signaling regulates both malignant cells and stromal cells of the tumor microenvironment (TME), constituting a potential therapeutic target against brain cancers. Here, we investigated the C–C chemokine receptor type 7 (CCR7) and the chemokine (C–C-motif) ligand 21 (CCL21) for their expression and function in human GBM and then assessed their therapeutic potential in preclinical mouse GBM models. In GBM patients, CCR7 expression positively associated with a poor survival. CCL21–CCR7 signaling was shown to regulate tumor cell migration and proliferation while also controlling tumor associated microglia/macrophage recruitment and VEGF-A production, thereby controlling vascular dysmorphia. Inhibition of CCL21–CCR7 signaling led to an increased sensitivity to temozolomide-induced tumor cell death. Collectively, our data indicate that drug targeting of CCL21–CCR7 signaling in tumor and TME cells is a therapeutic option against GBM.

**Supplementary Information:**

The online version contains supplementary material available at 10.1007/s00018-023-04788-7.

## Introduction

Malignant gliomas are the most common primary tumors of the CNS and comprise an extremely heterogeneous group of tumors of glial origin [1, 2]. Glioblastoma (GBM, WHO grade IV glioma) is the most frequent and aggressive type of tumor, accounting for more than 50% of gliomas, with poor patient survival and a lack of effective therapies [1–3]. GBMs are invasive, angiogenic, and proliferative tumors characterized by important cellular and molecular heterogeneity [3, 4]. Because of these characteristics, and despite aggressive treatment with surgical resection and radiotherapy with concurrent and adjuvant chemotherapy based on temozolomide (TMZ), virtually all patients experience recurrence and the overall survival rarely exceeds 20 months [4, 5]. Several novel approaches, such as anti-angiogenic agents (bevacizumab) and new chemotherapy protocols have been investigated, but unfortunately, those reaching clinical practice have achieved limited success [6–8]. More recently, clinical trials have investigated the use of checkpoint inhibitors and chimeric antigen receptor (CAR) T-cell therapy for the treatment of GBM but these treatments showed limited efficacy [9–12].

The GBM TME contains several different cell types which interact to promote resistance to current therapies through as yet incompletely understood mechanisms [13, 14]. Tumor-associated microglia and macrophages (TAMs) are the most abundant stromal cells in the GBM microenvironment, comprising up to 25% of the tumor mass [15–18]. TAMs promote the dysmorphic and aberrant tumor angiogenesis induced by GBM progression, which is characterized by aberrantly dilated blood vessels with perfusion defects, reduced branch points, and increased vessel leakage [13, 19–21]. Importantly, this aberrant tumor angiogenesis prevents the delivery of therapeutic agents and its mechanisms remain incompletely understood, although proangiogenic signaling molecules, including VEGF-A, angiopoietins and SLIT2 are known to be involved [19–22]. Recent studies have also highlighted the importance of TAMs in driving immunosuppression and therapy resistance [18, 23–25].

Several regulators of TAMs have been described [18], but most attempts to target these cells for GBM treatment have been unsuccessful [26, 27]. For example, the GBM microenvironment rapidly develops resistance to Colony stimulating factor 1 receptor (CSF-1R) inhibition, with alternative pathways compensating for its role in TAM activation [28]. It is therefore compelling to identify new regulators of TAMs for therapeutic targeting in combination with available chemotherapy treatments. In this respect, chemokines are attractive candidates as they are known to modulate the interplay between tumor cells and immune cells [29].

CC-chemokine ligand 21 (CCL21) regulates immune cell chemotaxis, homeostasis, and tolerance mechanisms [30]. CCL21 and CCL19 are the only known ligands for CC-chemokine receptor 7 (CCR7), which is a G protein-coupled 7 transmembrane chemokine receptor expressed by immune cells including macrophages, dendritic cells (DCs), B and T lymphocytes [30]. CCL21 and CCL19 together with CCL25 also bind to another membrane receptor, CC-X-chemokine receptor (CCX-CKR or CCRL1), which acts as a scavenger receptor [30]. CCR7 activation leads to Gα dependent MAPK (Erk1/2), PIK3, JAK and small GTPase activation in different cell types [31]. Despite their apparent redundancy in terms of CCR7 binding and signaling activation, only CCL21 is expressed in lymphatic endothelial cells [30] and in vivo ectopic CCL21 expression is a much stronger inducer of immune cell infiltration when compared to CCL19 [32].

The main function of CCL21-CCR7 signaling is to guide homing of immune cells to secondary lymphoid organs: CCL21 expression in lymphatic endothelial cells and high endothelial venules allows immune cells to migrate towards draining lymph nodes. Furthermore, CCL21 expression by fibroblast reticular cells in the lymph nodes allows immune cell migration and T cell differentiation [30].

Recently, CCL21-CCR7 signaling has emerged as a potential anti-tumor target. CCL21-CCR7 signaling activation has been associated with cancer cell invasion, metastasis and lymphangiogenesis in breast, prostate, head and neck and colon cancers, as well as B cell malignancies [33–39]. A metastatic mechanism was demonstrated in which CCR7-expressing tumor cells could ‘home’ to lymph-nodes like immune cells [40, 41]. CCL21 also participates in regulating the immune escape of melanoma by inducing macrophage and Treg recruitment into the TME [42]. In oral squamous cell carcinoma, CCR7 expression has been associated with macrophage recruitment and M2 polarization, and in vitro treatment of human macrophage cell lines with CCL21 recapitulates increased cell migration and M2 polarization [43]. However, literature on the role of CCL21 in GBM is limited. One study used commercial GBM cell lines to show that CCL21-CCR7 induced tumor cell invasiveness and epithelial-mesenchymal transition (EMT) in a TGFβ1 dependent manner in vitro [44]. CCL21 was also reported to be expressed in mouse GL261 tumor and stromal cells in vivo, and correlated to macrophage recruitment and tumor cell survival [45]. Finally, one last study evaluated the use of CCL21-coupled nanoparticles as an immunotherapy in mice carrying GBM tumors implanted in the periphery, which does not model a CNS TME properly [46]. Therefore, the role of CCL21-CCR7 pathway in GBM is not yet fully understood. Particularly, it remains unclear if this pathway is active in GBM patient samples and patient-derived cell lines and how it impacts the tumor microenvironment in preclinical models.

In the present study, we investigated the expression and function of CCL21-CCR7 in GBMs, through the analysis of GBM patient datasets, patient-derived cell lines, and by manipulating CCL21-CCR7 signaling in preclinical models of GBM.

## Results

### CCR7 expression increases with glioma grades and is associated with decreased patient survival

**Fig. 1:**
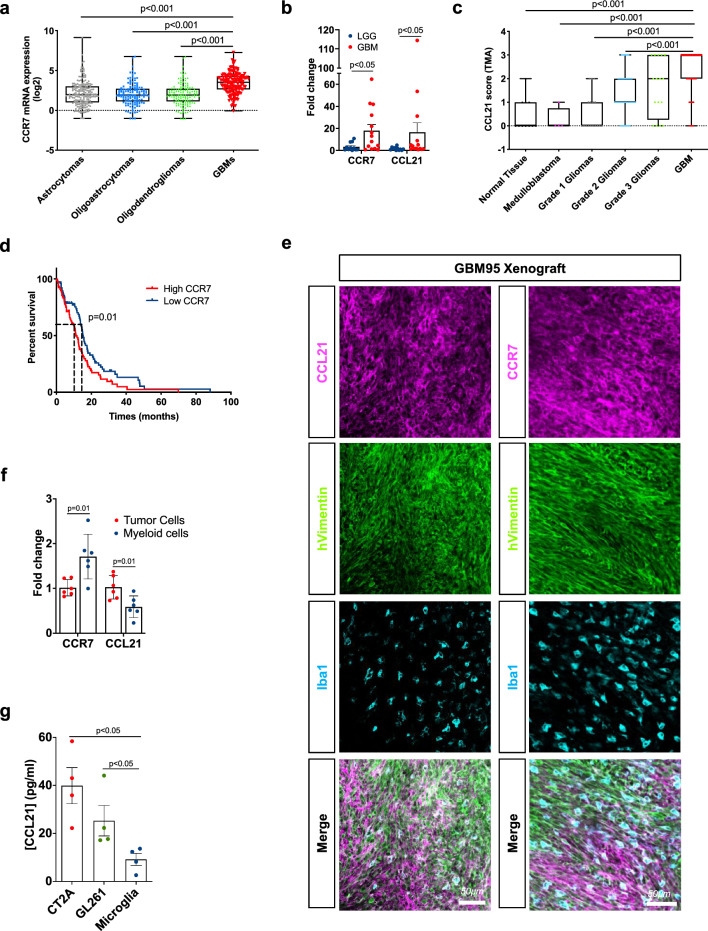
CCR7 expression increases with glioma grades and is associated with worse patient prognosis. **a** In silico analysis of glioma microarray data from TCGA patient database showing CCR7 expression in different glioma subtypes (n = 194 Astrocytomas, 129 Oligoastrocytomas, 129 Oligodendrogliomas and 152 GBMs; One-Way ANOVA). **b** CCR7 and CCL21 qPCR expression in glioma patient samples (Grade IV, n = 14, Grades I to II, n = 11, Mann–Whitney U test). **c** Tissue microarray (TMA) analysis showing CCL21 protein expression in different brain tumor subtypes (n = 11 Normal Brain Tissue, 4 Medulloblastomas, 17 WHO grade I gliomas, 42 WHO grade II gliomas, 16 WHO grade III gliomas, 75 WHO grade IV gliomas; One-Way ANOVA). **d** In silico analysis of TCGA glioblastoma RNAseq patient database (n = 78 high and 77 low CCR7 expressing patients; O.S., 11.2 months for high expression, 15.4 months for low expression, log-rank test). **e** Immunohistochemistry on sections of late-stage tumors generated from Gbm95 patient-derived cell line. Sections were stained for tumor cells (human Vimentin, green) and total TAMs (Iba1, cyan). CCL21 and CCR7 staining (magenta) demonstrate different cells from the tumor microenvironment expressing both proteins. **f** qPCR analysis of CCL21 and CCR7 expression in GFP^+^ tumor cells and myeloid cells (CD45^+^CD11b^+^CD3^−^) FACS-sorted from CT-2A mice glioblastomas (n = 6 independent tumors, day 21 after implantation, Mann–Whitney U test). **g** ELISA from conditioned medium from CT-2A and GL261 GBM cell lines and primary microglial cell to quantify CCL21 secretion (n = 4 independent cultures, Mann–Whitney U test). Data are presented as mean ± s.e.m

To investigate CCR7 expression in malignant gliomas in silico, we used multiple data sources from The Cancer Genome Atlas (TCGA) database (TCGA LGGGBM microarray and GBM RNAseq), a tissue microarray (TMA) of 153 primary CNS tumors and 11 normal brain samples, and the RNA database from a primary glioma patient cohort (25 patients, 14 GBMs and 11 LGGs). We observed increased CCR7 expression in GBM compared with all other low-grade glioma (LGG) subtypes (Fig. 1a). CCL21 expression was not detected in this dataset. By QPCR, we observed elevated expression levels of both CCR7 and CCL21 in a cohort of 14 WHO grade IV GBM compared to 11 LGG patients (WHO grade I, II, and III gliomas) (Fig. 1b, Table 1). By immunostaining, CCL21 expression was also higher in neoplastic samples than in normal tissues, and increased with glioma grades, with highest expression in WHO grade IV GBMs (Fig. 1c, Supplementary Fig. 1a, b, Table 2). TCGA RNA sequencing data from GBM patients also showed that high CCR7 expression was associated with reduced survival in these patients (overall survival: 11.2 versus 15.4 months for high and low expression respectively, Fig. 1d). CCL21 and CCR7 expression in human GBM tumors, therefore, correlates with glioma malignancy and patient survival.

**Table 1:** Patient characteristics of samples from the cohort of the Brain-Tumor-Imm-2014 study, used for qPCR analysis in Fig. 1b

	Number of patients	Gender		Average age at diagnosis	OS (months)
		Male	Female		
LGG	11	6	5	45.3 (25–71)	22
GBM	14	7	7	56 (26–76)	45.4
Total	25	13	12	51.5 (25–76)	31.75

**Table 2:** Patient characteristics of samples from the cohort of the Hospital Universitário Clementino Fraga Filho (HUCFF), used for TMA analysis in Fig. 1c

	Number of patients	Gender		Average age at diagnosis
		Male	Female	
Normal Tissue	11	6	5	43.3 (26–56)
Medulloblastoma	4	1	3	25.3 (16–36)
Grade 1 Glioma	17	9	8	29.8 (4–58)
Grade 2 Glioma	42	21	21	38.2 (13–74)
Grade 3 Glioma	16	6	10	30.3 (4–53)
GBM	75	31	44	58 (21–84)
Total	165	74	91	46.5 (4–84)

Orthotopic xenografts of human GBM cells into immunodeficient mice allowed us to further assess the expression of CCR7 and CCL21 proteins on sections of tumoral brain tissue with a predominant localization of CCR7 in Iba1^+^ TAMs (cyan), while CCL21 immunoreactivity localized predominantly in human vimentin^+^ tumor cells (green) but also in TAMs (Fig. 1e). Expression of CCR7 and CCL21 was also confirmed in different patient-derived and commercially available GBM cells (Supplementary Fig. 1c, d). To determine effects of pathway manipulation in mouse GBM models, we implanted green fluorescent protein (GFP)-expressing CT2A glioma tumor cells into C57BL/6 mice [19, 47]. 21 days after tumor cell inoculation, flow-cytometry combined with RT-qPCR analyses showed that *Ccr7* and *Ccl21* transcripts were detected in CD45^−^GFP^+^ CT2A tumor cells and CD45^+^CD11b^+^CD3^−^ myeloid cells (Fig. 1f), suggesting that CCL21 may activate tumor cells and TAMs via autocrine or paracrine signaling. Finally, CCL21 ELISAs performed on conditioned medium (CM) from CT-2A and GL261 GBM tumor cells or primary microglial cells confirmed higher CCL21 secretion from tumor cells when compared to microglia (Fig. 1g).

### CCL21-CCR7 signaling regulates microglia/macrophage chemotaxis

**Fig. 2:**
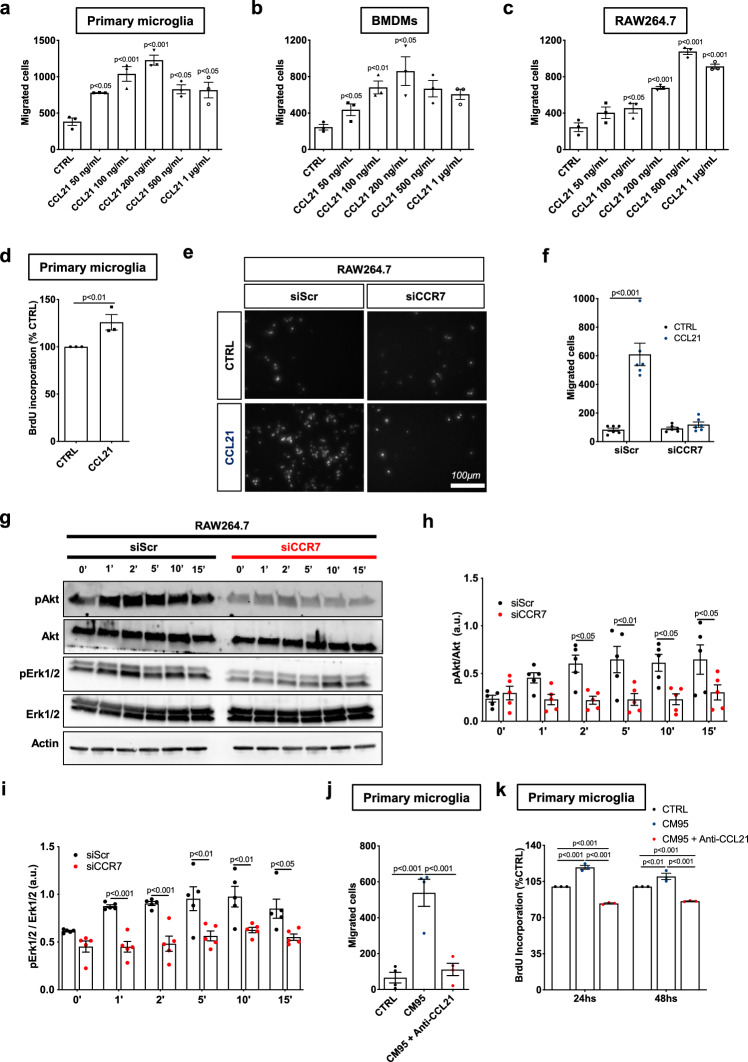
CCL21 induces microglia/macrophage migration and proliferation via CCR7. **a–c** Quantification of transwell assay of primary microglial cells (**a**), bone marrow derived macrophages (BMDM) (**b**) and RAW267.4 macrophages (**c**) in response to increasing concentrations of CCL21 (n = 3, One-way ANOVA, p values related to CTRL). **d.** Microglia proliferation measured by BrdU incorporation assay after treatment with 200 ng/mL of CCL21 for 24hs (n = 3, Mann–Whitney U test). **e** Transwell assay of RAW267.4 macrophages transfected with scramble (Scr) or CCR7 siRNA after CCL21 treatment (200 ng/mL). **f** Quantification of (**e**) (n = 6, Two-way ANOVA). **g** Western blot analysis of Akt and Erk1/2 phosphorylation induced by CCL21 (200 ng/mL) after treatment of RAW267.4 macrophages transfected with scramble (Scr) or CCR7 siRNA. **h**, **i** Quantification of western blots in (**g**) (n = 4 independent experiments, two-way ANOVA). **j** Quantification of transwell assay of primary microglia in response to Gbm95 Conditioned Medium (CM95) pre-incubated with control or CCL21 neutralizing antibodies (n = 4, One-way ANOVA). **k** Microglia proliferation measured by BrdU incorporation assay after treatment with CM95 pre-incubated with control or CCL21 neutralizing antibodies for 24 and 48 h (n = 3, Two-way ANOVA). Data are presented as mean ± s.e.m

We next investigated the role of CCL21-CCR7 signaling in the behavior of microglia/macrophages in vitro. Using transwell chamber migration assays with mouse primary microglia, bone marrow-derived macrophages (BMDM) and RAW264.7 macrophages, we showed that CCL21 in the bottom chamber chemoattracted all cell types in a dose-dependent manner (Fig. 2a-c, Supplementary Fig. 2a), and induced proliferation of microglial cells (Fig. 2d). CCR7 siRNA allowed us to knockdown *Ccr7* expression in RAW267.4 macrophages (Supplementary Fig. 2b-d) and abrogate macrophage migration towards CCL21 (Fig. 2e–f). CCR7 siRNA also inhibited CCL21-induced Akt and Erk1/2 phosphorylation in RAW264.7 macrophages (Fig. 2g–i). In a complementary experiment, we observed that CM derived from GBM 95 (GBM CM) in the bottom chamber induced microglial migration and that this effect was inhibited by GBM CM-pretreatment with a commercially available anti-CCL21 neutralizing antibody (CCL2-bAbs) (Fig. 2j). Neutralizing CCL21 in GBM CM also abrogated GBM-induced microglial proliferation (Fig. 2k). These data indicate that GBM-derived CCL21 induces microglia-macrophage chemoattraction and microglia proliferation in a CCR7-dependent manner.

### CCL21-mediated tumor supportive polarization of microglia/macrophages

**Fig. 3:**
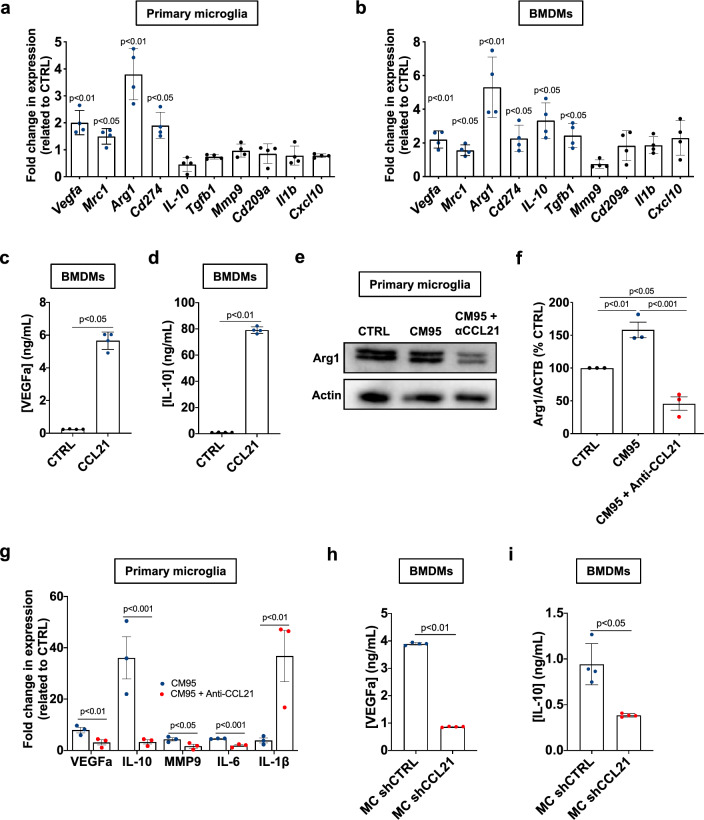
CCL21 induces tumor-supportive phenotype in microglia and macrophages. **a**, **b** qPCR analysis of *Vegfa, Mrc1, Arg1, Cd274, Il-10, Mmp9, Tgfβ, Cd209a, Il-*1*β* and *Cxcl10* in primary microglial cells (**a**) or BMDMs (**b**) 24 h after treatment with CCL21 (200 ng/mL) or control vehicle (PBS 0.5% BSA) treatment. Data show fold change compared to CTRL (n = 4, Mann–Whitney U test). **c**, **d** ELISA from conditioned medium from CCL21-treated BMDMs to quantify VEGFa (**c**) and IL-10 (**d**) (*n* = 4 independent cultures, Mann–Whitney U test)**. e.** Western blot for Arg1 in microglial cells after treatment with Gbm95 Conditioned Medium (CM95) pre-incubated with control or CCL21 neutralizing antibodies. **f** Quantification of (**e**) (n = 3, One-way ANOVA). **g** qPCR analysis of *Vegfa*, *Il-10*, *Mmp9*, *Il-6* and *Il-1β* in primary microglial cells 24 h after treatment with CM95 pre-incubated with control or CCL21 neutralizing antibodies (n = 3, One-way ANOVA). **h**, **i** ELISA from conditioned medium from BMDMs treated with CM from shCTRL and shCCL21a GL261 cells to quantify VEGFa (**h**) and IL-10 (**i**) (*n* = 4 independent cultures, Mann–Whitney U test)**.** Data are presented as mean ± s.e.m

Next, we tested whether CCL21 may mediate the activation and the tumor-supportive polarization of microglia/macrophages. Morphologically, CCL21-treated microglial cells exhibited a reduced cell area and a smaller number of protrusions when compared to control cells and to LPS-activated microglial cells (Supplementary Fig. 2e–g), suggesting CCL21 may trigger activation of microglial cells. With respect to the gene expression profile, CCL21 increased the expression of tumor-supportive genes such as *Vegfa, Mrc1*, *Arg1*, and *Cd274* (PD-L1) in microglia (Fig. 3a), as well as in BMDMs that also showed increased expression of *Il-10* and *Tgfβ1* (Fig. 3b) [16, 18, 48]. Using ELISA analysis of supernatants of primary macrophage cultures, we confirmed that CCL21 stimulated the production of VEGF-A and IL-10 (Fig. 3c-d). GBM CM-treated microglial cells showed similar increased expression of tumor-supporting proteins such as Arg1, that was abrogated by CCL21-blocking Abs (Fig. 3e, f). The expression of *Vegfa*, *Il-10*, *Il-6* and *Mmp9* transcripts detected under GBM CM treatment was also strongly reduced upon CCL21 neutralization, while microglial *Il-1β* expression was robustly increased (Fig. 3g). The differences in *Il-10* and *Il-1β* expression changes between recombinant CCL21 treatment and upon CCL21 neutralization from GBM CM suggests that CCL21 cooperates with other soluble factors secreted by tumor cells to induce TAM polarization in the TME.

ELISA analysis of macrophage culture supernatants provided convergent data, showing that GBM tumor cells treated with shCCL21 siRNA to knockdown *Ccl21* produced less VEGF-A and IL-10 than shCTRL-transduced GBM cells (Fig. 3h, i). Finally, we also characterized the cytokine expression profile of GBM CM-treated microglial cells using cytokine arrays. Microglial cells treated with CM from shCTRL-transduced GBM expressed high levels of angiopoietin 2, IGFBP6, CD142, LIF, NGAL (MMP9), CSF-1, MMP2, CCL5, Col18a1, TNFRSF11B, and CXCL2. In contrast, microglial cells exposed to CCL21-depleted GBM CM upregulated the production of IL-1α and CXCL16 (from shCCL21-transduced GBM) (Supplementary Fig. 3). Interestingly, microglial cells treated with CM from shCCL21 knockdown tumor cells downregulated cytokines associated with angiogenesis (angiopoietin 2, MMP9, MMP2, Col18a1), tumor invasiveness (MMP9 and MMP2) and tumor-supportive TAMs (CSF-1, CCL5, CXCL2) when compared to microglia treated with CM from shCTRL cells. These data indicate that CCL21 promotes tumor-supportive microglial cell polarization [13, 16, 18]. Furthermore, IGFBP6 has been associated with GBM tumor cell chemoresistance, tumor-supportive TAM polarization and immune escape [49–51]. Taken together, these data indicate that CCL21-CCR7 signaling drives TAM behavior by promoting their recruitment to the TME and their tumor-supportive phenotype, thereby stimulating tumor growth.

### CCL21-CCR7 signaling promotes GBM cell migration, proliferation, and resistance to TMZ

**Fig. 4:**
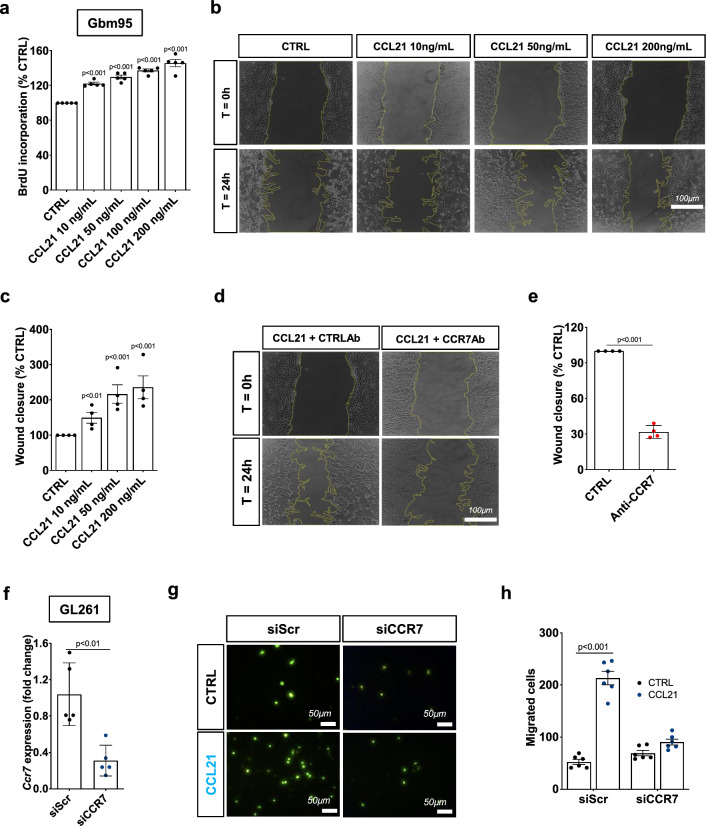
CCL21 induces tumor cell proliferation and migration.** a** Gbm95 proliferation measured by BrdU incorporation assay after treatment with increasing concentrations of CCL21 for 24hs (n = 5 independent cultures, One-way ANOVA). **b** Gbm95 scratch wound migration in response to increasing concentrations of CCL21. **c** Quantification of wound closure shown in (**b**) (n = 4 independent experiments, Mann–Whitney U test). **d** Gbm95 scratch wound migration in response to CCL21 (200 ng/mL) after pretreatment with control or CCR7 neutralizing antibodies. **e.** Quantification of wound closure shown in (**d**) (n = 4 independent experiments, Mann–Whitney U test). **f** qPCR analysis of CCR7 siRNA in cultured GL261 glioma cells 48 h after transfection (n = 5, Mann–Whitney U test). **g.** Transwell assays of GL261 glioma cells transfected with or without CCR7 siRNA. **h** Quantification of (**g**) (n = 6, Two-way ANOVA). Data are presented as mean ± s.e.m

To investigate the response of GBM cells to CCL21 signaling, we exposed CCR7-expressing human and murine GBM cell lines to CCL21 or PBS/BSA control. CCL21 increased proliferation and scratch wound migration of human GBM95 cells in a dose-dependent manner (Fig. 4a–c). Murine GL261 GBM cells were also chemoattracted by CCL21 in a dose-dependent manner (Supplementary Fig. 4a, b). The CCL21-induced migration of GBM95 cells was inhibited by commercially available CCR7 neutralizing antibodies (Fig. 4d, e). Furthermore, CCL21 added to the bottom chamber of a Boyden transwell attracted murine GL261 GBM cells in a CCR7 dependent manner (Fig. 4f–h). Mechanistically, CCL21 induced Erk1/2 and Akt phosphorylation in tumor cells and CCR7 knockdown abrogated Akt and Erk1/2 activation (Supplementary Fig. 4c–e). This data demonstrates that CCR7 signaling is important for GBM tumor cell migration and proliferation, as previously described for other types of tumors [35, 36, 39–41].

**Fig. 5:**
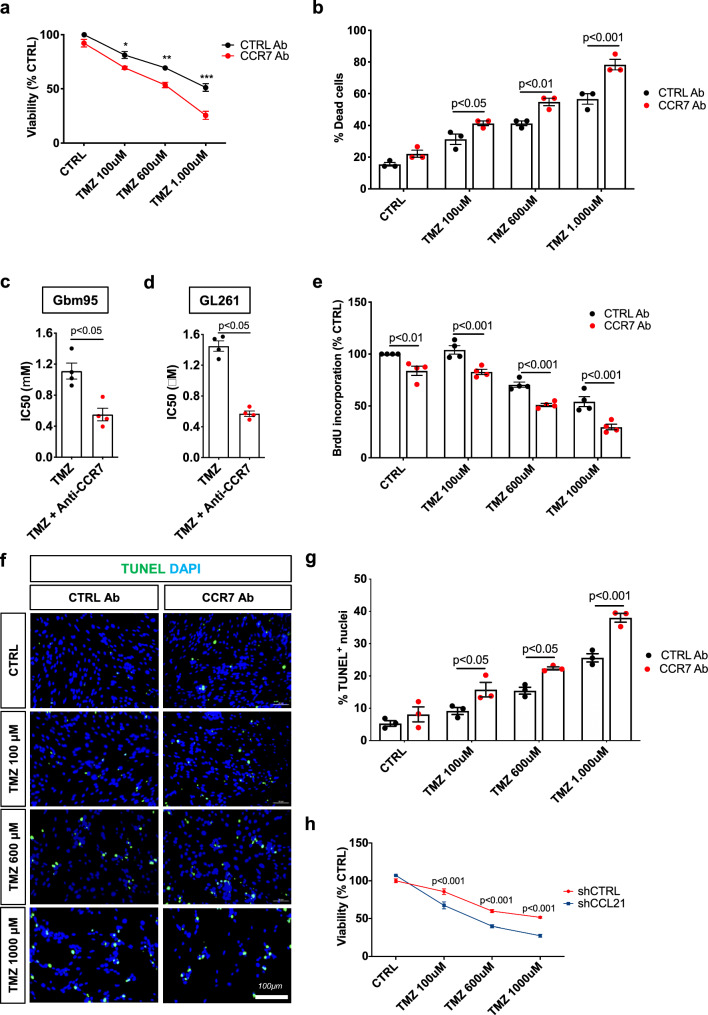
CCR7 blocking antibodies sensitizes GBM cells to Temozolomide treatment. **a**, **b** Gbm95 viability measured by MTT (**a**) and cell death measured by counting with trypan blue (**b**) 24 h after treatment with increasing concentrations of Temozolomide (TMZ) combined with control or CCR7 neutralizing antibodies (n = 3 independent experiments, One-way ANOVA). **c** **d** IC50 of TMZ combined with control or CCR7 neutralizing antibodies for Gbm95 (**c**) and GL261 (**d**) cell lines (n = 4 independent experiments, IC50 = 1110 μM for TMZ and 550.6 μM for TMZ + anti-CCR7 for Gbm95 and IC50 = 1449 μM for TMZ and 570.3 μM for TMZ + anti-CCR7 for GL261, Mann–Whitney U test). **e** Gbm95 proliferation measured by BrdU incorporation assay after treatment with increasing concentrations of TMZ combined with control or CCR7 neutralizing antibodies for 24 h (n = 4 independent experiments, One-way ANOVA). **f** Gbm95 apoptosis measured by TUNEL immunostaining after treatment with increasing concentrations of TMZ combined with control or CCR7 neutralizing antibodies. **g** Quantification of (**f**) (n = 3 independent experiments, One-way ANOVA). **h** In vitro shCTRL and shCCL21a GL261 glioma cell viability in response to TMZ treatment (n = 4, two-way ANOVA). Data are presented as mean ± s.e.m

We next tested whether GBM cell survival and resistance to chemotherapy involved CCL21-CCR7 signaling. CCR7 inhibition using neutralizing antibodies alone did not reduce Gbm95 or GL261 cell viability, however, CCR7 inhibition increased GBM cell sensitivity to chemotherapy with TMZ, which is the current gold standard therapy for GBM (Fig. 5a, b, Supplementary Fig. 5a, b). The combined treatment of GBM cells with TMZ and CCR7 blocking antibodies (CCR7-bAbs) reduced the TMZ IC_50_ by 50% (Fig. 5c, d). Moreover, CCR7-bAbs suppressed tumor cell proliferation, alone, and even more when combined with TMZ (Fig. 5e). The blockade of CCR7 signaling by CCR7-bAbs did not induce GBM cell apoptosis (TUNEL^+^ assay) although it potentiated the apoptotic effect of TMZ (Fig. 5f, g). Similar effects were observed using siRNAs to knockdown CCR7 (Supplementary Fig. 5c–g). Finally, we also observed that shCCL21 potentiated the toxic effect of TMZ on GBM cell survival (Fig. 5h). These findings suggest that blocking CCR7 signaling in GBM cells may improve the effect of TMZ-based chemotherapy against GBMs.

### *Ccl21* knockdown in GBM normalizes tumoral vessels and leverages TMZ survival effect

To generate a syngeneic mouse model of CCL21-deficient GBM, we first knocked down *CCL21* in GL261 and CT-2A GBM cells by lentiviral transfection of GFP-coupled *CCL21a-*targeting shRNA and FACS-sorted for GFP^+^ cells, which resulted in a 50% reduction of *Ccl21* transcript expression (Supplementary Fig. 6a, b). GBM tumor cell spheroids were then inoculated into the brain of ROSAmT/mG reporter mice [52]. In these mice, all stromal cells in the TME constitutively express membrane tdTomato fluorescent protein, which allows longitudinal examination of tumor angiogenesis and immune cell recruitment using in vivo 2-photon microscopy [19, 20].

**Fig. 6:**
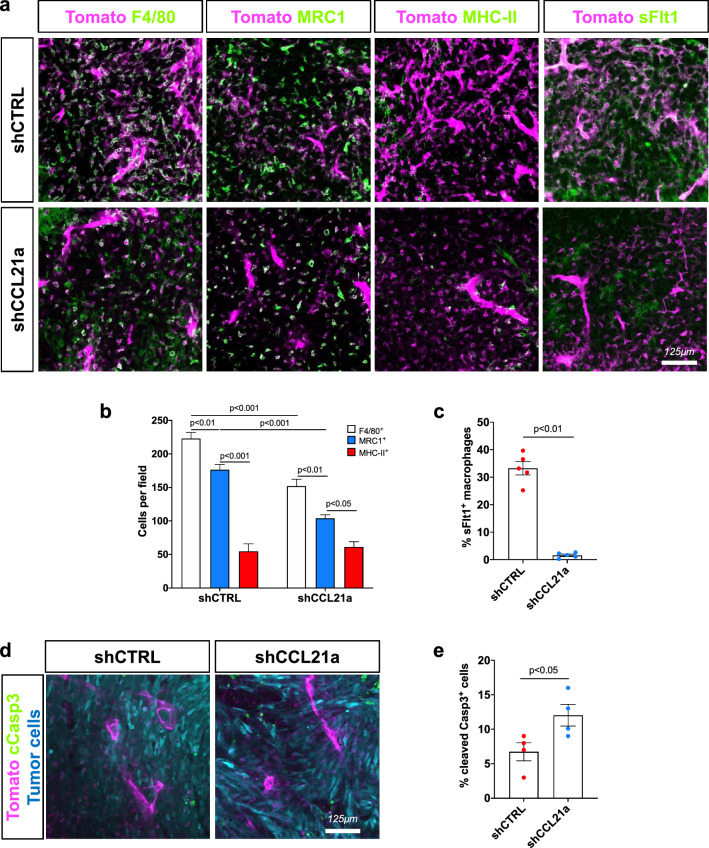
CCL21 promotes TAM recruitment and VEGF production in mouse glioma.** a** Immunohistochemistry on sections of late stage shCTRL or shCCL21a GL261 tumors (day 30 after tumor implantation). In magenta we show tdTomato^+^ stromal cells in the TME and in green labeling for total TAMs (F4/80), activated MHC-II^+^ antigen-presenting cells (APCs), MRC1(CD206)^+^ tumor-supportive cells and VEGFa expression using sFlt1 binding. **b**. Quantifications corresponding to (**a**) (n = 5 mice per group, 5 fields per tumor, Two-Way ANOVA). **c** Quantification of soluble-Flt1 binding to sections of GL261 shCTRL and shCCL21a tumors (n = 5 mice per group, 5 fields per tumor, Mann–Whitney U Test). **d** Anti-cleaved Caspase3 staining on sections of late stage GL261 shCTRL or shCCL21a tumors. tdTomato^+^ stromal cells in the TME are shown in magenta, GFP^+^ tumor cells in blue and cleaved caspase 3 staining in green. **e** Quantification of cleaved caspase 3^+^ GFP^+^ tumor cells from (**d**) (n = 4 mice per group, 5 fields per tumor, Mann–Whitney U Test). Data are presented as mean ± s.e.m

Post-mortem histology showed that the number of F4/80^+^ total TAMs and MRC1^+^ tumor-supportive TAMs in the TME were decreased by *Ccl21a* knockdown in GBM, while activated MHC-II^+^ antigen-presenting cells (APCs) were unchanged (Fig. 6a-b, Supplementary Fig. 6c, d). We evaluated VEGF-A production in the TME using an in vitro sFlt1 binding assay that showed reduced TAM labeling in *Ccl21a* knockdown GBM in comparison with controls (Fig. 6c). shCCL21a-transduced GBM also had an increased number of cleaved caspase 3^+^ apoptotic cells when compared to shCTRL GBM (Fig. 6d, e).

**Fig. 7:**
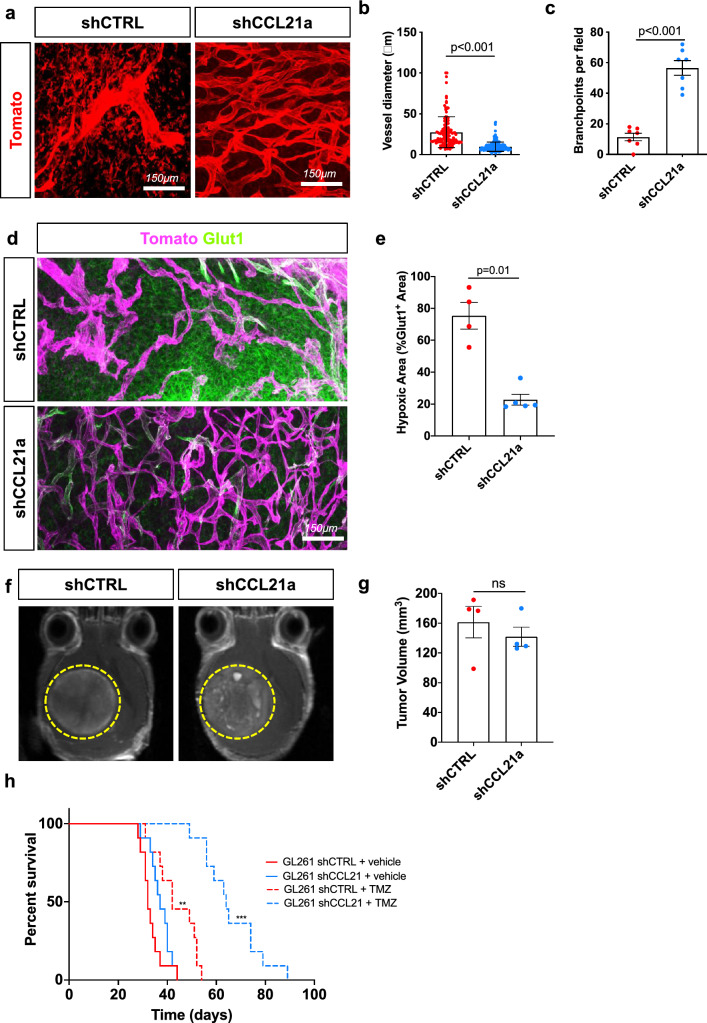
CCL21 knockdown normalizes vasculature and increases response to TMZ treatment. **a** In vivo two-photon images of ROSA^mTmG^ mice bearing late stage GL261 shCTRL or shCCL21a tumors (day 30 after tumor implantation). **b**, **c** Quantification of vessel diameter (**b**) and branchpoints (**c**) from (**a**) (n = 6 mice per group, Mann–Whitney U Test). **d**, **e** Glut1 immunohistochemistry and quantifications of Glut1 + hypoxic areas in the tumor (n = 6 mice per group, Mann–Whitney U Test). In magenta we observe stromal cells in the tumor microenvironment (tdTomato^+^) and in green Glut1 staining. **f** T1-weighted MRI images of shCTRL and shCCL21a GL261 tumors 30 days after tumor spheroid implantation. **g.** Quantification of tumor size from MRI images on (**f**) (n = 4 tumors per group). **h** Experimental survival trial design: 8-week-old mice were engrafted with GL261 shCTRL or shCCL21a cells and randomly assigned to vehicle or temozolomide (TMZ) treatment (40 mg/kg) (n = 11 mice per group, O.S. = 32 days for shCTRL, 42 days for shCTRL + TMZ, 37 days for shCCL21a, 64 days for shCCL21a + TMZ; Multiple comparisons log-rank test). Data are presented as mean ± s.e.m

In vivo two-photon imaging demonstrated that blood vessel morphology was altered by *CCL21a* knockdown in GBM cells as their diameter was decreased and their ramification was increased compared to the tumoral blood vasculature of mice bearing shCTRL-transduced GBM (Fig. 7a–c). Using glucose transporter 1 (Glut1) immunostaining to detect both hypoxic areas within the tumor mass and active glucose transport in blood vessels, we found that mice with shCCL21a-transduced tumors displayed less hypoxic brain areas and more Glut1 coverage of blood vessels compared to mice bearing shCTRL-transduced GBM, indicating a benefit on blood–brain barrier function (Fig. 7d, e, Supplementary Fig. 6e).

To assess GBM tumor growth in live mice, we used post-gadolinium T1-weighted MRI imaging that showed no difference in size between shCCL21a- and shCTRL-transduced GBM at 30 days after intracerebral implantation (Fig. 7f, g). The overall survival was also similar between mice bearing shCCL21a- and shCTRL-transduced GBM (median survival: 37 days for shCCL21a and 32 days for shCTRL; Fig. 7h). In contrast, the depletion of *Ccl21a* expression in GBM potentialized the benefit of TMZ chemotherapy as shown by the prolonged survival of mice with shCCL21-transduced GBM compared to shCTRL-transduced GBM bearing mice (median survival: 64 days for shCCL21a + TMZ and 42 days for shCTRL + TMZ; Fig. 7h).

## Discussion

This work provides new mechanistic information on CCL21-CCR7 signaling in GBM tumor growth and reveals pathway regulation of paracrine interactions between tumor cells and TAMs. We observed pro-migration and survival effects of CCL21-CCR7 signaling on GBM cells, which are characteristically invasive and resistant to therapy. One major problem in the care of GBM patients is the early occurrence of tumor relapse owing to its invasive nature [3, 4]. CCR7 inhibition has been shown to be an important factor for tumor cell invasiveness in several different cancer types [33–36, 40–42], and our data suggest that this is also true for GBM. Thus, inhibiting CCR7 to target tumor cell invasiveness and prevent tumor cell migration may prove to be an interesting strategy to prevent GBM relapse.

Another characteristic of GBM is its resistance to cytotoxic chemotherapy [5]. Temozolomide-based chemotherapy has been the gold standard for GBM patients over the past 15 years because of its ability to cross the blood–brain barrier (BBB) and to diffuse in the CNS at sufficiently high concentrations. Nevertheless, tumor cell resistance to TMZ and non-uniform perfusion by dysmorphic tumor blood vessels limit its efficacy. Here, we showed that knockdown of CCL21 in GL261 GBM tumor cells prolonged mouse survival in the presence of TMZ-based chemotherapy, whereas TMZ alone was less effective in prolonging mouse survival, and CCL21 knockdown alone had no significant effect on survival or tumor size. We show that tumor cell intrinsic and extrinsic processes are involved in CCL21 dependent enhancement of the TMZ response. CCL21 knockdown GBM cells and anti-CCR7 treated GBM cells are more sensitive to TMZ induced cell death and proliferation inhibition. Moreover, CCL21-CCR7 pathway inhibition normalized the vasculature, which may increase TMZ access to the tumor and enhance tumor cell killing. Vascular normalization is well known to improve tumor perfusion and thereby increase chemotherapy delivery and efficacy, as observed in preclinical models and in GBM patients [53]. Vascular normalization also occurred in CCL21 knockdown tumors without TMZ treatment, without reducing tumor size. In this setting, the vascular normalization in the TME may in fact accelerate tumor growth, as shown previously in GBM and other tumors [20, 54, 55], thereby balancing the negative effect of reduced CCL21-CCR7 signaling in tumor cells.

The GBM microenvironment is characterized by recruitment of tumor-supportive TAMs, depletion of T lymphocytes and dysmorphic angiogenesis, which cooperate to promote the aggressive behavior of GBM cells [4, 13]. TAM abundance in the GBM microenvironment contributes to immunosuppression and vascular dysmorphia in the TME by secreting growth factors, immunomodulatory and pro-angiogenic cytokines [13, 19, 20, 23, 56–58]. TAMs are a heterogeneous population comprising recruited and activated microglial cells and infiltrating monocyte-derived macrophages from the periphery [48, 59, 60]. Recent GBM single cell transcriptomics studies have suggested that these cells have different polarization profiles, with monocyte-derived TAMs being more associated with immune suppression and poor prognosis, whereas microglia-derived TAMs present a more pro-inflammatory profile [48, 59, 60]. Interestingly, we also observed differences in CCL21-induced activation of microglia and monocyte-derived macrophages in vitro, with an increased expression of immunosuppressive cytokines IL-10 and TGFβ induced by CCL21 in BMDMs but not in microglial cells. This different response of microglial cells and macrophages to CCL21 suggests that this chemokine could be important for the differential activation of microglia-derived and monocyte-derived TAMs in the context of brain tumors. In vivo single cell transcriptomic analysis of microglia and monocyte TAMs under CCL21 inhibition will allow better understanding of the differential impact of CCL21-CCR7 signaling in microglia- or monocyte-derived TAMs during GBM development.

The process of TAM recruitment and polarization involves multiple molecular signals such as CSF-1, Slit2, CCL2, IL-6 and OPN [61]. Attempts to target only one signaling pathway, for example by CSF-1R inhibition, have so far failed to provide long-term efficacy as tumors develop resistance to therapy [26, 28], justifying the search for novel targets. Here, we demonstrated that CCL21/CCR7 signaling in TAMs is important to drive their tumor-supportive polarization in the TME and promote tumor growth and should therefore be considered as a potential new therapeutic target that could be combined with approaches to target other important regulatory pathways such as CSF-1 or Slit2.

The present evidence of CCL21-CCR7 signaling role in GBM progression, through promoting tumor cell proliferation/survival and the tumor-supportive behavior of TAMs, therefore indicate CCL21-CCR7 blocking drugs as new opportunities to normalize the TME, reduce resistance to chemotherapy treatment*,* and thus improve anti-GBM chemotherapy.

## Materials and methods

### Study approval and mice

All in vivo experiments were conducted in accordance with the Brazilian Experimental Animal Use Guidelines and the European Community for Experimental Animal Use Guidelines (L358-86/609EEC). The protocols were approved by the Ethical Committee of the Health Sciences Center of the Federal University of Rio de Janeiro (n° 0001/16) and by the French Ministry of Higher Education, Research, and Innovation (n°MESRI23570). Animals were housed with free access to food and water under a 12 h light/dark cycle. Male C57BL/6 J and C57Bl6 ROSA^mT/mG^ mice (8 to 10 weeks old) were purchased from the Jackson Laboratory.

### Human brain tumor samples

Frozen tumor samples were obtained from 25 patients at the Catholic University of Leuven after obtaining informed consent and approval by the UZ Leuven ethical committee for the Brain-Tumor-Imm-2014 study. The study BRAIN-TUMOR-IMM-2014 (S57028) on human tissue was reviewed and approved by the Ethics Committee Research UZ/KU Leuven (Herestraat 49, 3000 Leuven, Belgium) on 08 SEP 2014. These samples consisted of materials in excess of those required for diagnostic purposes and pathological classification was based on central review of the histopathology of patients. All participants provided written informed consent prior to participation in the study.

The Tissue Microarray (TMA) study required 153 brain tumor cases and 11 normal brain tissue samples selected from the archive of the Pathology Service of Clementino Fraga Filho Hospital, Federal University of Rio de Janeiro. All cases were independently reviewed by 2 neuropathologists and classified according to the WHO classification for brain tumors from 2007. The 78 glioma diagnoses were as follows: medulloblastoma (4 cases), WHO grade 1 ganglioglioma (8 cases), WHO grade 1 dysembryoplastic neuroepithelial tumors (DNET, 5 cases), WHO grade 1 astrocytoma (3 cases), WHO grade 1 subependymoma (1 case), WHO grade 2 astrocytoma (10 cases), WHO grade 2 oligoastrocytoma (8 cases), WHO grade 2 oligodendroglioma (13 cases), WHO grade 2 ependymoma (10 cases), WHO grade 3 astrocytoma (6 cases), WHO grade 3 oligodendroglioma (10 cases), and primary WHO grade 4 glioblastoma (75 cases). One site of interest was marked on a slide for each case, and the corresponding area was extracted from the respective paraffin blocks. A 1.0 mm cylinder was then extracted from the donor block, corresponding to the most representative area of the case, using the manual method with custom-built hypodermic needles. Hematoxylin–eosin (H-E) slides were obtained using a microtome, as well as 5 µm sequential sections for staining.

### Cell lines

Human tumor cell lines Gbm95, Gbm02, and Gbm11 were established in our laboratory [62, 63]. The use of patients’ surgical specimens for the establishment of cell lines for in vitro and in vivo research provided written informed consent from the patients and was approved by the Brazilian Ministry of Health Ethics Committee under the Institutional Review Board (IRB—Research Ethics Committee of Hospital Universitário Clementino Fraga Filho) consent CEP-HUCFF No. 002/01. The T98G glioma cell line was acquired from the American Type Culture Collection (ATCC, Manassas, VA, USA).

Cell lines were grown and maintained in DMEM-F12 supplemented with 10% fetal bovine serum (FBS). Culture flasks were maintained at 37 °C in a humidified 5% CO2 and 95% air atmosphere. Cells displaying exponential growth were detached from the culture flasks with 0.25% trypsin/ethylenediaminetetraacetic acid (EDTA) and seeded. All cultured GBM cell lines were immunoreactive for GFAP, vimentin, and nestin, but were not labeled with IB4 (data not shown). The macrophage cell line RAW264 and murine GBM cell lines GL261 and CT-2A were cultured in the same manner, and all experiments were performed between five passages.

### Bioinformatic analysis

For ‘The Cancer Genome Atlas’ (TCGA) datasets, TCGA-GBMLGG microarray data of 688 glioma patients and RNAseqV2 normalized data of 151 primary glioblastoma multiforme and associated clinical data were downloaded from the cBioPortal and GlioVis data portals (https://gliovis.bioinfo.cnio.es and https://www.cbioportal.org/study/summary?id=gbm_tcga) [64]. For survival analysis, in each dataset the cohort was split into two groups of patients defined by the mean level of target gene expression. Overall survival (in months) was used to estimate survival distributions using the Kaplan–Meier method, and the distributions were compared using the log-rank test.

### Tissue microarray (TMA) analysis

Immunohistochemistry on brain tumor cases and normal brain tissue samples was performed using an anti-CCL21 primary antibody (Abcam) in the tissue microarray block. A PowerVision + Poly-HRP IHC Detection System (Leica) was used according to the manufacturer’s instructions. Two neuropathologists analyzed the slides and ranked CCL21 expression as follows: 0 (no expression), 1 + (< 25% of the tissue positive for staining), 2 + (25–75% of the tissue positive for staining), or 3 + (> 75% of the tissue positive for staining).

### Human brain tumor RNA extraction

RNA was purified from frozen tissue samples in liquid nitrogen using the RNeasy kit (Qiagen). 0.5 μg of RNA was reverse transcribed using SuperScript IV reverse transcriptase and random primers (Invitrogen). Quantitative PCR was performed as described for sorted cells from the tumor microenvironment using Quantitec qPCR primers (Qiagen). The data were first normalized to the actin level in each sample, and the relative expression levels of the different genes were calculated using the comparative Ct method.

## Surgery procedures and GBM cell inoculation

Craniotomy and glioblastoma spheroid implantation were performed as previously described [47]. Briefly, a 5-mm circle was drilled between the lambdoid, sagittal, and coronal sutures of the skull of ketamine/xylazine-anesthetized C57Bl6 ROSA^mT/mG^ mice. A 250-μm diameter CT-2A or GL261 glioblastoma cell spheroid was injected into the cortex and sealed with a glass coverslip cemented on top of the mouse skull. 21 (for CT-2A) or 30 days (for GL261) after tumor implantation, anesthetized mice were transcardially perfused with 2% PFA solution. The Mouse brains were harvested and fixed overnight in 4% PFA) at 4 °C. For immunohistochemistry, the brains were washed with PBS and sectioned using a vibratome (200um-400 μm sections).

Xenografts from GL261 cells for survival experiments and patient-derived cells were performed as previously described [19, 65]. For Temozolomide (Sigma) treatment, mice were injected intraperitoneally with 40 mg/kg in 0.2 mL on days 7, 11, 15, and 19 after tumor implantation.

### Live imaging

For multiphoton excitation of endogenous fluorophores in experimental gliomas, we used a Leica SP8 DIVE in vivo imaging system equipped with 4tune spectral external hybrid detectors and an InSightX3 laser (SpectraPhysics). The microscope was equipped with an in-house designed mouse-holding platform for intravital imaging (stereotactic frame, Narishige; gas anesthesia and body temperature monitoring/control, Minerve). Excitation of ROSAmT/mG reporter mice was performed at 1040-nm fixed wavelength and at 925-nm wavelength for Green Fluorescent Protein (GFP) signal from genetically modified tumor cells.

### Isolation and qPCR analysis on GBM cells and TAMs

Ketamine/xylazine-anesthetized tumor-bearing mice received trans-cardiac perfusion with 30 ml of ice-cold PBS. Tumors were harvested and incubated in DMEM medium containing 2.5 mg/ml collagenase D and 5 U/ml DNase I for 20 min at 37 °C. The digested tissue was passed through a 40 μm nylon cell strainer (Falcon), and red blood cells were lysed (red blood cell lysis buffer, Merck).

After blocking with mouse FcR blocking reagent (MACS Miltenyi Biotec), cells were stained with the following monoclonal antibodies: anti-CD45 Alexa Fluor 594 (R&D Systems), anti-CD11b BV450 (BD) and anti-CD3 PE/Cy5 (BioLegend) antibodies. TAMs (CD45^+^CD11b^+^CD3^−^) and tumor cells (GFP^+^ CD45^−^) were sorted on BD FACS Aria II. The cells were then shock-frozen in liquid nitrogen and stored at -80 °C until further use. Total RNA was isolated using a NucleoSpin RNA XS kit (Macherey–Nagel). Real-time quantitative PCR (qPCR) reactions were performed in duplicate using the MyIQ real-time PCR system (Bio-Rad) with iQ SYBR Green Supermix (Bio-Rad) and QuantiTect qPCR primers (Qiagen, Table 3). Each reaction contained 10 ng cDNA and 250 nM forward and reverse primers. Fold changes were calculated using the comparative CT method.

**Table 3:** List of qPCR Primers used in this study

Primer	Cat No
Mm_ACTB_1_SG	QT00095242
Mm_CCR7_1_SG	QT00240975
Mm_MRC1_1_SG	QT00103012
Mm_VEGFA_1_SG	QT00160769
Mm_MMP9_1_SG	QT00108815
Mm_TGFB1_1_SG	QT00145250
Mm_IL1B_2_SG	QT01048355
Mm_CXCL10_1_SG	QT00093436
Mm_ARG1_1_SG	QT00134288
Mm_IL10_1_SG	QT00106169
Mm_PDCD1IG1_1_SG	QT00148617
Mm_Ccl21a_1_SG	QT00284753
Mm_CD209A_1_SG	QT00116312
Mm_Il6_1_SG	QT00098875
Hs_ACTB_1_SG	QT00095431
Hs_CCL21_1_SG	QT00202692
Hs_CCR7_1_SG	QT00045507

### Immunofluorescence labeling

Vibratome sections were blocked and permeabilized in TNBT buffer (0.1 M Tris pH 7.4; NaCl 150 mM; 0.5% blocking reagent from Perkin Elmer, 0.5% Triton X-100) overnight at 4 °C. Tissues were then incubated with primary antibodies anti-F4/80 (Life Technologies, 1:100), anti-MRC1 (R&D Systems, 1:100), anti-MHCII (Thermo Scientific, 1:100), anti-Glut1 (Millipore, 1:200), anti-cleaved Caspase 3 (Abcam, 1:200), anti-Iba1 (Wako, 1:100), anti-cleaved caspase 3 (Abcam, 1:300), anti-CCR7 (R&D Systems, 1:100), or anti-CCL21 (R&D Systems, 1:100) diluted in TNBT overnight at 4 °C, washed in TNT buffer (0.1 M Tris pH 7.4; NaCl 150 mM; 0.5% Triton X-100) at least seven times, and incubated with appropriate Alexa Fluor 488, 555 or 647 conjugated antibody (Life Technologies, 1:400) diluted in TNBT overnight at 4 °C. Samples were then washed at least seven times in TNT and mounted on slices in fluorescent mounting medium (Dako). Images were acquired using a Leica SP8 inverted confocal microscope. For quantifications of stromal cell numbers, individual cells were defined based on the tdTomato expression. When antibody staining co-occurred with tdTomato expression, this was considered a positive cell.

### Soluble Flt-1 binding assay

To detect VEGFa expression, vibratome sections were blocked and permeabilized in TNBT overnight at 4 °C. Tissues were then incubated with 1 μg/ml recombinant mouse soluble Flt-1 FC chimera (R&D Systems) diluted in TNBT for 6 h at room temperature. Samples were rinsed three times in TNT and fixed in 4% PFA for 3 min. Samples were washed at least 7 times in TNT and incubated in Alexa Fluor 647 coupled anti-human IgG secondary antibodies (Life Technologies, 1:200) diluted in TNBT overnight at 4 °C. Tissues were washed at least 7 times and mounted on slides in fluorescent mounting medium (Dako). Images were acquired using a Leica SP8 inverted confocal microscope.

### Primary cell cultures

Primary cell cultures of bone marrow-derived macrophages (BMDMs) were derived from C57BL/6 mice by flushing the femurs and tibias with PBS. Bone marrow cells were resuspended in DMEM GlutaMax (Gibco) containing 1% penicillin/streptomycin (Gibco), 20% Fetal Bovine Sereum (FBS; Gibco), and 100 ng/mL M-CSF (R&D Systems). Cells were incubated overnight at 37 °C and 5% CO2 in non-treated bacterial dishes for adhesion of bone-marrow resident macrophages, and then changed to treated plastic dishes and cultured for 6 days with medium change every 2 days. Before the experiments, the cells were starved overnight in serum- and CSF-free media.

Primary cell cultures of microglial cells were derived from cortex of newborn C57BL/6 mice as previously described [66]. For morphological analysis, qPCR analysis, western blotting, and protein arrays, isolated primary cells were starved overnight in serum-free medium and then treated for 24 h with recombinant CCL21 or tumor cell conditionate mediums (CMs) alone or in combination with anti-CCL21 antibodies (Abcam, 8ug/mL). Proteome Profiler Mouse XL Cytokine Arrays (R&D Systems) were performed according to the manufacturer’s instructions using supernatant from 3 different cultures.

### ELISA

CCL21, VEGFa and IL-10 concentrations in conditioned medium (CM) from cells were determined by the sandwich ELISA method using Mouse CCL21/6Ckine, Mouse VEGF and Mouse IL-10 Quantikine ELISA Kits (R&D Systems) according to the manufacturer’s instructions.

### shRNA transfection of cultured cells

siRNA against mice and human CCR7 was purchased from Origene (Rockville, MD, USA) and transfection was carried out using siTran1.0 Transfection Reagent (Origene, Rockville, MD, USA) according to the manufacturer’s instructions. Briefly, 10^6^ cells were plated with 150uL of siRNA complexes overnight in Opti-MEM. The medium was replaced with complete DMEM before the analysis. All experiments were performed 48hs post-transfection.

### Wound-healing assay

The wound healing assay was performed as previously described, with minor modifications [67]: Briefly 1.5 × 10^5^ tumor cells were seeded on 24 well plates and after 8hs, the cells were treated with Mitomycin C overnight to prevent proliferation. Cell monolayers were then scraped into straight lines with a p10 pipette tip, and debris was removed by washing the monolayers with fresh serum-free culture medium. Images were captured using a Nikon Eclipse T300 microscope before treatment with CCL21 and after 24 h of treatment. ImageJ software (v1.46; National Institutes of Health, Bethesda, MD, USA) was used to analyze the images, and the scratched areas were determined using the “Polygon Selection Tool” for each time-point and treatment. The results were normalized to the scratched areas at 0 h. For the wound-healing assay with the CCR7 neutralizing antibody, cells were incubated for 15 min with 5 μg/mL anti-CCR7 antibodies before the addition of CCL21.

### Transwell assay

To evaluate chemotactic migration of GBM cells or macrophages in the direction of CCL21, Transwell assays were performed:1.0 × 10^5^ cells were plated in the top chambers in 150 μL of serum-free medium, with increasing concentrations of CCL21 (from 50 to 1000 ng/mL) or GBM conditioned medium in the bottom chambers. Cells were incubated overnight at 37 °C and 5% CO_2_, the medium was removed from both chambers, and inserts were fixed with 70% ethanol for 20 min at room temperature before staining with Giemsa or live stained with Calcein AM (Thermo Fisher). Then, the wells were washed, and 10 pictures per well were acquired at 10 × magnification using a Nikon Eclipse T300 epifluorescence microscope. Migrated cells per field were counted using the ImageJ software.

### Viability assay

GBM cells were treated for 24 h with different concentrations of Temozolomide (TMZ) alone or in combination with 5 μg/mL anti-CCR7. The viability of GBM cells was determined by trypan blue staining. Briefly, after 24hs treatment, cells were detached from 24-well plates with 0.5 mM EDTA in PBS, centrifuged, and resuspended in 50uL of fresh medium. Then, a 1:1 dilution with 0.4% Trypan Blue solution was made, and viable (unstained) and non-viable cells (blue) were counted using a hemocytometer. The curve for the calculation of the TMZ concentration necessary to inhibit cell proliferation by 50% was determined using GraphPad Prism 5 (version 5.00; GraphPad Software, Inc., San Diego, CA, USA).

### BrdU incorporation assay

GBM cells or primary microglia were plated in 96 well plates and treated for 24 h or 48 h. The proliferation capacity of the cells was determined by quantifying BrdU incorporation into the DNA of replicating cells using a Cell Proliferation ELISA kit according to the manufacturer’s instructions (Cell Proliferation ELISA for BrdU, Roche). Briefly, cells treated for 24 h were incubated with BrdU labelling solution (0.1 μl/ml) for 120 min at 37 °C in a humidified atmosphere (5% CO_2_). Next, the cells were incubated with FixDenat solution and anti-BrdU POD (anti-BrdU-FLUOS) according to the manufacturer’s instructions (Roche). Colorimetric analyses were performed using a VICTOR X3 multilabel plate reader, and absorbance was determined at 450 nm (Perkin-Elmer, Waltham, MA, USA).

### TUNEL assay

GBM cells were seeded on round glass coverslips in 24 well plates and treated for 24 h with different concentrations of TMZ alone (100, 600 μM and 1.000 μM) or in combination with 5 μg/mL anti-CCR7. The cells were then fixed in 4% paraformaldehyde for 30 min at room temperature, followed by permeabilization with 0,025% Triton-X100 in PBS for 30 min. For the detection of apoptotic cells, the Cick-iT TUNEL Alexa Fluor 488 Imaging Assay Kit was used according to the manufacturer’s instructions. For siRNA-transfected cells, coverslips were then blocked with 5% BSA in PBS for 30 min and incubated with mouse anti-Ki67 (1:400, Dako) primary antibody at 4 °C overnight. Next, the cells were washed with PBS and incubated with anti-mouse Alexa564 secondary antibody at 37 °C for 1 h. Finally, nuclei were counterstained with DAPI and images were captured using a Nikon Eclipse T300 microscope, and ImageJ software (v1.46; National Institutes of Health, Bethesda, MD, USA) was used to count total, proliferating, and apoptotic cells.

### RT-qPCR on cultured cells

After siRNA transfection and/or treatment, RNA from RAW264.7 or primary cells were purified using a RNeasy-kit (Qiagen). RNA (1000 ng) was reverse transcribed using SuperScript II reverse transcriptase and random primers (Invitrogen). Quantitative PCR was performed as described for sorted cells from the tumor microenvironment using Quantitec qPCR primers (Qiagen). The data were first normalized to the actin level in each sample, and the relative expression levels of the different genes were calculated using the comparative Ct method.

### Immunolabeling of cultured cells

GBM cells were seeded on round glass coverslips in 24-well plates and cultured until they reached the desired confluence. To detect CCL21 and CCR7 expression, the cells were fixed with 4% paraformaldehyde, blocked with 5% BSA in PBS for 30 min at room temperature, and incubated with rabbit anti-CCL21 (1:200, Abcam) and mouse anti-CCR7 (1:100, R&D Systems) primary antibodies at 4 °C overnight. Next, the cells were washed with PBS and incubated with specific Alexa secondary antibodies at 37 °C for 1 h. Nuclei were stained with DAPI. Slides were examined using an SP8 inverted confocal microscope (Leica Microsystems).

### Immunoblotting analysis

For protein phosphorylation analysis, cells were starved in serum-free medium overnight before treatment with 200 ng/mL CCL21 alone or in combination with anti-CCR7 antibodies for determinate time points. Cells were lysed in RIPA lysis buffer containing phosphatase and protease inhibitors (Roche). Equal amounts of proteins were separated on a 4–15% Criterion precast gel (Bio-Rad) and transferred onto a nitrocellulose membrane with Transblot Turbo (Bio-Rad). Membranes were blocked with 5% non-fat milk in TBS-T for 30 min at room temperature and incubated with primary antibodies against CCR7 (R&D Systems, 1:500), CCL21 (Abcam, 1:1.00), actin (Sigma, 1:4000), anti-phospo p44/42 MAP kinase (phospho-ERK, Cell Signaling, 1:1000), anti-p44/42 MAP kinase (total ERK, Cell Signaling, 1:1000), anti-pAkt Ser473 (Cell Signaling, 1:1000), and anti-Akt (Cell Signaling, 1:1000) overnight at 4 °C with agitation. After washing with TBS-T, the membranes were incubated with the appropriate HRP-conjugated secondary antibodies for 3 h at room temperature under agitation. Western blots were developed with a chemiluminescence HRP substrate (Bio-Rad) on a luminescent image analyzer, ChemiDoc XRS + (Bio-Rad). Densitometric analyses were performed using ImageJ 1.49v software (Wayne Rasband, National Institutes of Health, Bethesda, MD, USA).

### Statistical analysis

For continuous variables, data are presented as mean ± s.e.m. between-group comparisons were performed using the Mann–Whitney U test or t-test, depending on the sample size for continuous variables. In cases where more than two groups were compared, a one-way ANOVA test was performed, followed by Tukey’s multiple comparison test, and the results were considered significantly different if p < 0.05. For comparisons involving grouped data, a two-way ANOVA test was performed, followed by Tukey’s multiple comparison test, and the results were considered significantly different if p < 0.05. All the analyses were performed using Prism 6.0 software (GraphPad).

## Supplementary Information

Below is the link to the electronic supplementary material.

Supplementary Figure 1. Tissue Microarray (TMA) analysis shows increased expression of CCL21 in glioma patient samples. a. Descriptive table of the samples analyzed using TMA. Two neuropathologists analyzed the slides and ranked CCL21 expression as follows: 0 (no expression), 1+ (<25% of the tissue positive for staining), 2+ (25% to 75% of the tissue positive for staining), or 3+ (>75% of the tissue positive for staining). b. Representative images of WHO grades II/III and IV tumors with different intensities of CCL21 staining. c-d. Immunocytochemistry (c) and western blot analysis (d) of CCL21 and CCR7 in patient-derived (Gbm95, Gbm02, and Gbm11) and commercial (T98G) GBM cell lines. Supplementary Figure 2. CCL21 microglia/macrophage migration and activation. a. Transwell assay of primary microglial cells, bone marrow derived macrophages (BMDM) and RAW267.4 macrophages in response to increasing concentrations of CCL21 from Figure 2a-c. b. qPCR analysis of CCR7 siRNA in cultured RAW267.4 macrophages 48 hours after transfection (n=4, Mann-Whitney U test). c-d. Western blot analysis and quantification of CCR7 expression in RAW264.7 macrophages 48hs after transfection with control or CCR7 siRNA (n-4, Mann-Whitney U test). e. Representative images of primary microglia after control, LPS (1ug/mL) or CCL21 (200ng/mL) treatment. f-g. Cell area (f) and protrusions per cell (g) quantified after 24 h of treatment (n=4, 400 analyzed cells, one-way ANOVA). Data are presented as mean ± SEM. Data are presented as mean ± SEM. Supplementary Figure 3. CCL21 treatment changes the protein expression profile of microglial cells. Proteome Profiler Mouse XL Cytokine Array and quantification showing effects on microglial protein expression after treatment with conditioned medium (CM) from GL261 shCTRL or shCCL21a cells. Supernant from 3 individual cultures was used for profiling. Data are presented as mean ± SEM. Supplementary Figure 4. CCL21 induces GL261 glioma cell migration through CCR7. a. Transwell assay of GL261 glioma cells in response to increasing CCL21 concentrations. b. Quantification of (a) (n=4, one-way ANOVA). c. Western blot analysis of Erk1/2 and Akt phosphorylation induced by CCL21 (200ng/mL) after treatment of GL261 glioma cells transfected with scramble (Scr) or CCR7 siRNA. d-e. Quantification of western blots is shown in (c) (n=3 independent experiments, two-way ANOVA). Data are presented as mean ± SEM. Supplementary Figure 5. CCR7 inhibition sensitizes tumor cells to Temozolomide treatment by increasing apoptosis and decreasing proliferation. a-b. GL261 glioma cell viability (a) and cell death (b) 24 h after treatment with increasing concentrations of Temozolomide (TMZ) combined with control or CCR7 neutralizing antibodies (n=3 independent experiments, one-way ANOVA). c. qPCR analysis of CCR7 siRNA in cultured Gbm95 cells 48 h after transfection (n=6, Mann-Whitney U test). d. Gbm95 proliferation measured by Ki67 immunostaining after treatment with increasing concentrations of Temozolomide (TMZ) in scramble (Scr) or CCR7 siRNA-transfected cells. e. Quantification of (d) (n=3 independent experiments, One-way ANOVA). f. Gbm95 apoptosis measured by TUNEL immunostaining assay after treatment with increasing concentrations of Temozolomide (TMZ) in scrambled (Scr) or CCR7 siRNA-transfected cells. g. Quantification of (f) (n=3 independent experiments, one-way ANOVA). Data are presented as mean ± SEM. Supplementary Figure 6. CCL21 promotes TAM recruitment and polarization in mouse glioma. a-b. CCL21a qPCR expression in GL261 (a) and CT-2A (b) shCCL21a and shCTRL (n=4, Mann Whitney U Test). c. Immunohistochemistry of sections of late-stage shCTRL or shCCL21a CT-2A tumors (21 days after tumor implantation) for Glut1, total TAMs (F4/80), activated MHC-II+ antigen-presenting cells (APCs) and MRC1(CD206)+ tumor-supportive cells. d. Quantification of TAMs markers in (c) (n=4 mice per group, 5 fields per tumor, two-way ANOVA). e. Quantification of Glut1+ hypoxic areas in the tumors from (c) (n=6 mice per group, Mann-Whitney U test). Data are presented as mean ± SEM. Supplementary Figure 7. Model of CCL21-CCR7 signaling in the GBM microenvironment. Proposed model of how CCL21-CCR7 signaling in GBM tumor cells and TAMs affects GBM microenvironment

## Data Availability

The datasets analyzed during the current study are publicly available in the TCGA repository and were downloaded from the cBioPortal and GlioVis data portal repositories (https://gliovis.bioinfo.cnio.es and https://www.cbioportal.org/study/summary?id=gbm_tcga).
